# Neuralgic Amyotrophy with Cervical Root and Cranial Nerves Involvement in a Child 

**Published:** 2019

**Authors:** Mahmoud Reza ASHRAFI shrafi, Alireza TAVASOLI, Masood GHAHVECHI AKBARI

**Affiliations:** 1 Pediatrics Center of Excellence, Department of Pediatric Neurology, Children’s Medical Center, Tehran University of Medical Sciences, Tehran, Iran.; 2 Physical Medicine and Rehabilitation Department, Children’s Medical Center, Tehran University of Medical Sciences, Tehran, Iran

**Keywords:** Neuralgic amyotrophy, Child, Electromyography, Nerve conduction study

## Abstract

Idiopathic neuralgic amyotrophy (INA) is a disorder presented with acute severe pain in the upper extremity, followed by muscle weakness, paralysis and atrophy. INA is rare in children and few reports are found in the literature. Here, we report a case of INA in an 8-yr old boy from Iran following pharyngitis.

## Introduction

Idiopathic neuralgic amyotrophy (INA) (also called Parsonage-Turner syndrome or brachial plexus neuritis) is a distinct clinical syndrome which presents with acute severe pain in the upper extremity, shoulder or arm, lasting for several days or weeks, followed by muscle weakness, paralysis and atrophy (1,2). The precise mechanism is unknown, autoimmune pathogenesis due to intrinsic or extrinsic conditions are possible (1). Upper respiratory tract infections, immunizations, trauma were considered as possible etiology (3).

Reports of children with brachial neuritis (neuralgic amyotrophy or NA) with cranial nerves involvement in NA is rare (4-6). 

Here, we report a case of INA in an 8-yr old boy following pharyngitis. 

## Case presentation

An 8-yr old boy was admitted to Children’s Medical Center, Tehran University of Medical Sciences, Tehran, Iran, in 2016 with the diagnosis of bacterial pharyngitis with symptoms of sore throat, headache and fever. He was treated with intramuscular penicillin G benzathine 1200000 unit. After two days, agitation and sleep disturbance due to severe pain in right shoulder and arm were reported. The patient was treated with different analgesics. Oral acetaminophen was given for two weeks. After 6 d, the pain was gradually reduced and after two weeks, the patient was pain-free (measured by Wong-Baker FACES Pain Rating). Muscle weakness, dysphagia, asymmetry, and weakness in the facial muscles occurred. Cranial Neuropathies occurred 6 d after plexopathy. 

There was no family or previous history of a similar complaint.

**Figure 1:**
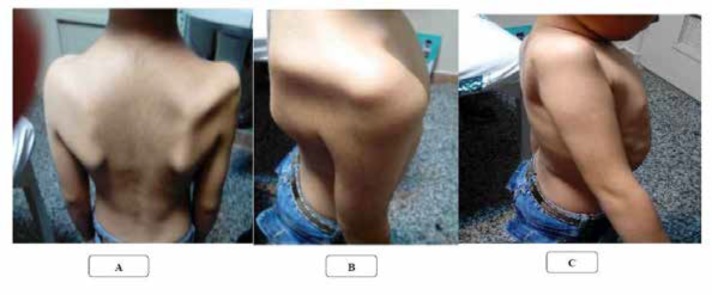
A: Atrophy of right trapezius, infraspinatus and supraspinatus muscles. B: Atrophy of right deltoid muscle. C: Right shoulder abduction weakness

On physical examination, muscle force of right arm in abduction was 0/5, in shoulder flexion and extension: 1/5, shoulder elevation: 3/5, elbow flexion and extension: 1/5, wrist flexion and extension: 4/5, forearm supination 2/5 and pronation 3/5, flexor digitrum profound and superficial: 4/5, intrinsic muscles of hand: 4/5 and neck flexion and extension: 2/5. There was weakness of right facial muscles upper and lower part (peripheral facial paresis). Extraocular muscles and pupils’ light reflex were normal on both sides. Other limbs had normal force. Sensory exam was normal. There were reduced deep tendon reflexes in the right upper limb. We observed atrophy of supraspinatus, infraspinatus, and deltoid muscles in the right side (Figure 1A, B, C). Gag reflex was reduced in the right and was normal in the left.

Blood lab tests including CBC diff, liver function test, thyroid function test, BUN, creatinine, ESR, CRP, urine analysis were measured which yielded neutrophil dominant leukocytosis and increased ESR. Throat culture was positive for *Streptococcus* group A and ASO titer was 700 IU/ml. Serologic tests for neurotrophic organisms including *Borrelia*, neurotropic viruses (HSV, VZV, HIV), *Bartonella* and hepatitis E were performed which all came negative. Lumbar puncture was not performed, as we did not have parents’ consent. 

Infectious diseases were the top of our differential diagnosis that all possible causes were evaluated. Paraneoplastic presentations were other possible diagnosis ruled out by oncology consultation. Guillain-Barré syndrome was also considered as another differential diagnosis, but asymmetrical presentation of symptoms and unilateral involvement were against the diagnosis. 

**Figure 2:**
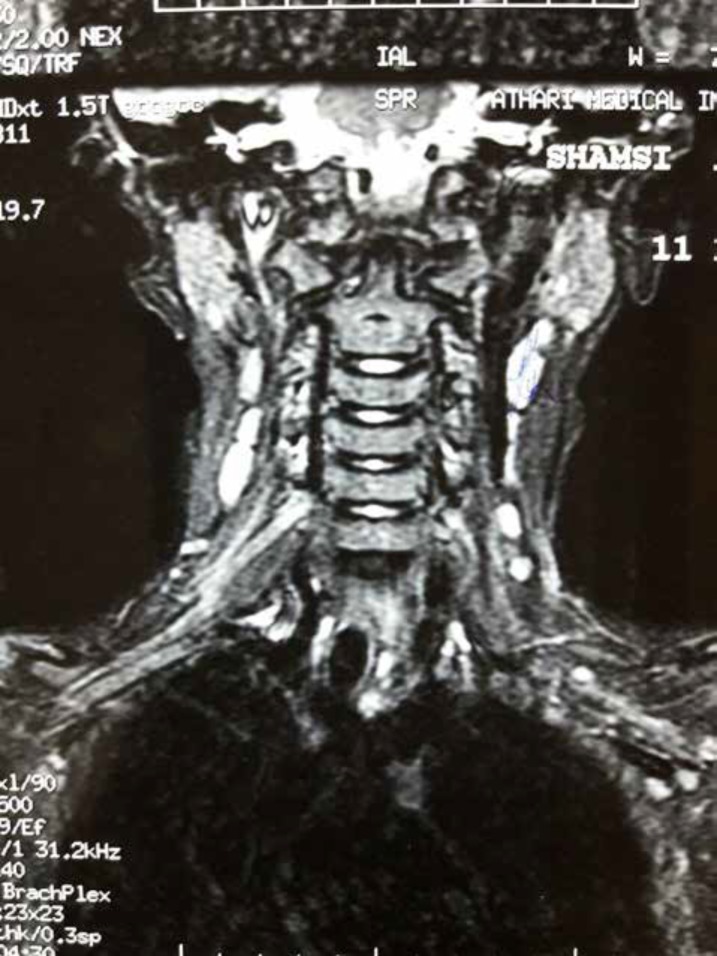
Cervical MRI showing root hypertrophy and enhancement in the right side (C4-C8), with more hypertrophy and enhancement at C4-C6 root

Right shoulder MRI was performed which yielded normal findings. The patient also underwent brain, cervical and brachial plexus MRI. Brain MRI was normal. Brachial plexus was normal, while in the cervical MRI, there was root hypertrophy and enhancement in the right side (C4-C8), with more hypertrophy and enhancement at C4-C6 root (Figure 2).

Electrodiagnostic study was performed 3 wk after symptoms initiation. Before the study, the temperature of the upper limbs was 31 centigrade. The sensory potential performed in an antidromic manner. Electrodiagnostic study showed normal SNAPs and F-waves with low amplitude or unobtainable CMAPs of right upper limb. Needle exam was neurogenic with active denervation (Table 1). These findings were compatible with an axonal type pre-ganglionic lesion at right C5-T1 territories, which were severe in C5-C7 levels and moderate at C8-T1 levels as well as partial axonal lesion of right accessory and facial nerves.

With the diagnosis of INA, patient received prednisolone 1 mg/kg/d for two weeks and physical therapy 3 times/week, the patient showed significant improvement in the muscle force of all muscle with no change in deltoid, supraspinatus and infraspinatus muscles. 

The patient was followed every 2 months. Improvement in cranial neuropathies occurred in 6 months and after 1 year; there was significant improvement in upper right limb muscles, except deltoid, infra and supraspinatus muscles.

**Table 1:** Nerve conduction studies (NCV) and electromyography (EMG) findings of the patient


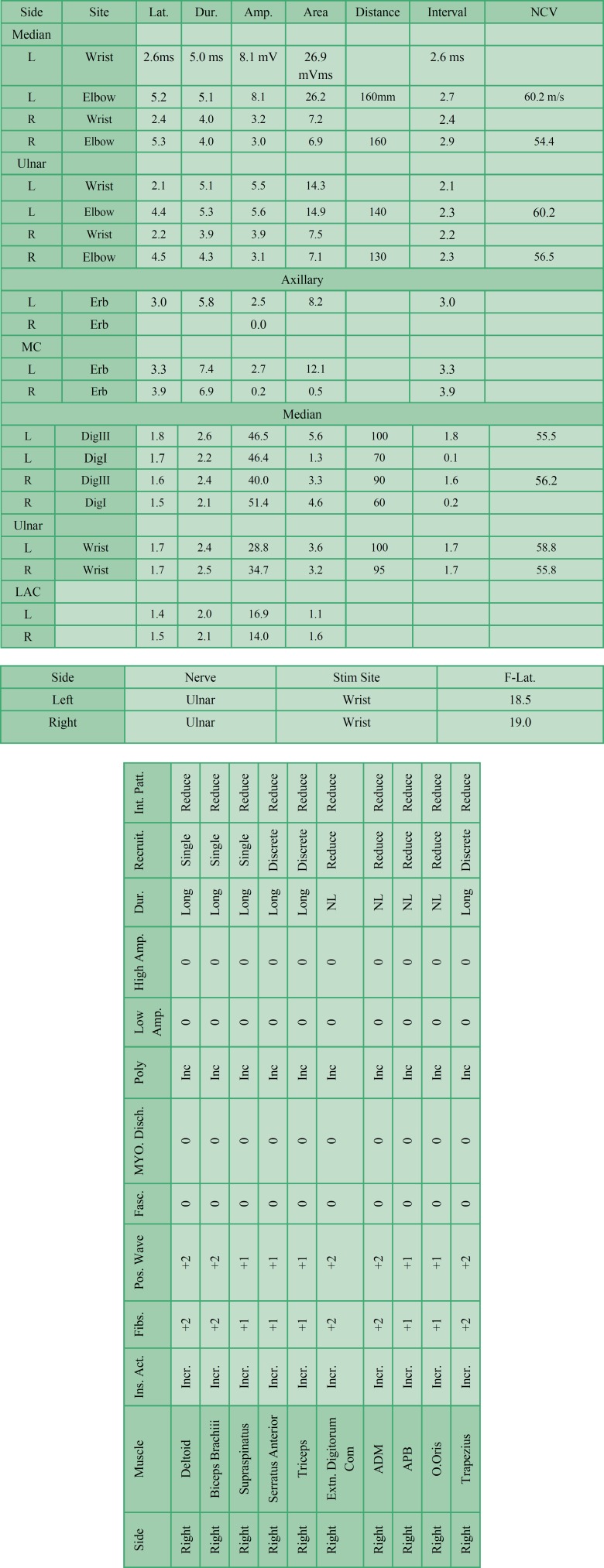


## Discussion

The common triad of INA is extreme pain at the onset, rapid paresis and atrophy of the upper arm, and a slow recovery that occurs over months to years (1). This disorder usually occurs in adults and there are only few cases reported in children (4-6). 

The exact etiology of INA is unknown; however, an immune-mediated response triggered by trauma, upper respiratory infections, stress and immunizations could be the cause (7). The symptoms in our case presented following pharyngitis. INA could occur in association with antibiotics. INA was reported in a 22 yr old male with cystic fibrosis after vancomycin administration (8), as well as in a 30 months old female child with acute respiratory distress and after cefoperazone and sulbactam injection (4). It is possible that in our case, the symptoms were triggered by antibiotics; however, there was no concise evidence to consider or rule out this possibility. In the case of cystic fibrosis (8), he was given vancomycin following confirmed bacterial respiratory infection. Although antibiotics were the cause for INA, the infections should also be considered. 

MRI is usually used to rule out other shoulder pain related pathologies. Nerve conduction studies (NCV) and electromyography (EMG) studies are the best modality to confirm a diagnosis of INA and may show axonal injury, demyelination, and spontaneous muscle activity. The common findings are abnormal sensory potentials, lack of paraspinal denervation potentials, and abnormal conduction velocities). These studies should perform 2-3 wk after the initiation of the disease (9). Sensory studies are normal in 80% of cases (10).

The main treatment principle for NA is supportive management, including analgesics, immunizations, and physiotherapy. Corticosteroids are recommended to reduce the duration of symptoms (9). However, no specific treatment has been proven to reduce neurologic impairment or improve prognosis. 

**In conclusion,** INA is rare in children especially when it accompanies with neurological findings. If diagnosed properly and differential diagnosis are excluded, it should be treated with analgesics, immunizations, and physiotherapy. 

## References

[1] van Alfen N (2011). Clinical and pathophysiological concepts of neuralgic amyotrophy. Nat Rev Neurol.

[2] Stutz CM (2010). Neuralgic amyotrophy: Parsonage-Turner Syndrome. J Hand Surg Am.

[3] van Alfen N, van Engelen BG The clinical spectrum of neuralgic amyotrophy in 246 cases. Brain2006.

[4] Jain S, Bhatt GC, Rai N, Bhan BD (2014). Idiopathic brachial neuritis in a child: A case report and review of the literature. J Pediatr Neurosci.

[5] Høst C, Skov L (4). Idiopathic neuralgic amyotrophy in children. Case report,.

[6] To WC, Traquina DN (1999). Neuralgic amyotrophy presenting with bilateral vocal cord paralysis in a child: a case report. Int J Pediatr Otorhinolaryngol.

[7] van Alfen N (2007). The neuralgic amyotrophy consultation. J Neurol.

[8] Finstad K, Guajardo JR, Scoville C (2008). Neuralgic amyotrophy associated with antibiotic therapy. AnnPharmacother.

[9] Yu DK, Cho YJ, Heo DH, Hong MS, Park SH (2010). Neuroradiologic and neurophysiologic findings of neuralgic amyotrophy. Korean Neurosurg Soc.

[10] van Alfen N, Huisman WJ, Overeem S, van Engelen BG, Zwarts MJ (2009). Sensory nerve conduction studies in neuralgic amyotrophy. Am J Phys Med Rehabil.

